# Desert salt flats as oases for the spider *Saltonia incerta* Banks (Araneae: Dictynidae)

**DOI:** 10.1002/ece3.1242

**Published:** 2014-09-20

**Authors:** Sarah C Crews, Rosemary G Gillespie

**Affiliations:** 1 Department of Environmental Science, Policy, & Management, UC Berkeley130 Mulford Hall, Berkeley, 94720-3114, California

**Keywords:** American southwest, dispersal, endemism, fragmentation, phylogeography, refugia

## Abstract

The deserts of southwestern North America have undergone dramatic changes over their recent geological history including large changes in size and connectivity during the Pleistocene glaciopluvial cycles. This study examines the population history of the rare spider *Saltonia incerta,* once thought to be extinct, to determine the role of past climatological events in shaping the structure of the species. This species is restricted to salt crusts of intermittent or dry lakes, streams or rivers in the desert southwest, a region that was much wetter during glacial periods. We examine the distribution and genetic variability of populations to test whether there is recent dispersal throughout the range of the species. Analyses of mitochondrial and nuclear DNA indicate significant population structure, with one major clade comprising New Mexico localities and one comprising California-northern Baja California localities. Finer-scale structure is evident within the California clade, although not all of the subclades are reciprocally monophyletic. However, isolation with migration analysis suggests that migration is very low to non-existent. These results extend the known distribution of *Saltonia*, provide genetic evidence of strong isolation among localities within drainage basins and between drainage basins and provide a mechanistic understanding of population connectivity after the aridification of the American southwest. The implication is that although the species' distribution has been fragmented, populations have persisted throughout this area, suggesting that desert salt flats may have served as refugia for at least some terrestrial species.

## Introduction

Throughout the Quaternary period, the entire ecosystem of southwestern North America changed dramatically in response to glacial climate change, volcanism and tectonic activity. One of the most extreme examples of change that occurred in this region is the transformation of wetter woodland and scrub habitats to some of the driest deserts on earth, including the iconic Death Valley. These geological and climatic shifts altered the distributional ranges of the region's biota (Grismer 1994; Masta 2000; Riddle et al. 2000; Hunter et al. 2001; Zink et al. 2001; Crews and Hedin 2006; Douglas et al. 2006; Castoe et al. 2007). Today, the effects of desertification can be seen in a number of unique environments in this area, such as sand dunes and dry lake beds, which frequently harbor endemic organisms (Andrews et al. 1979; Pavlik 1989). These species are not only of high conservation concern because of their limited distributions, but they are of considerable interest because their specific habitat requirements make them excellent indicators of past environmental changes.

**Figure 1:**
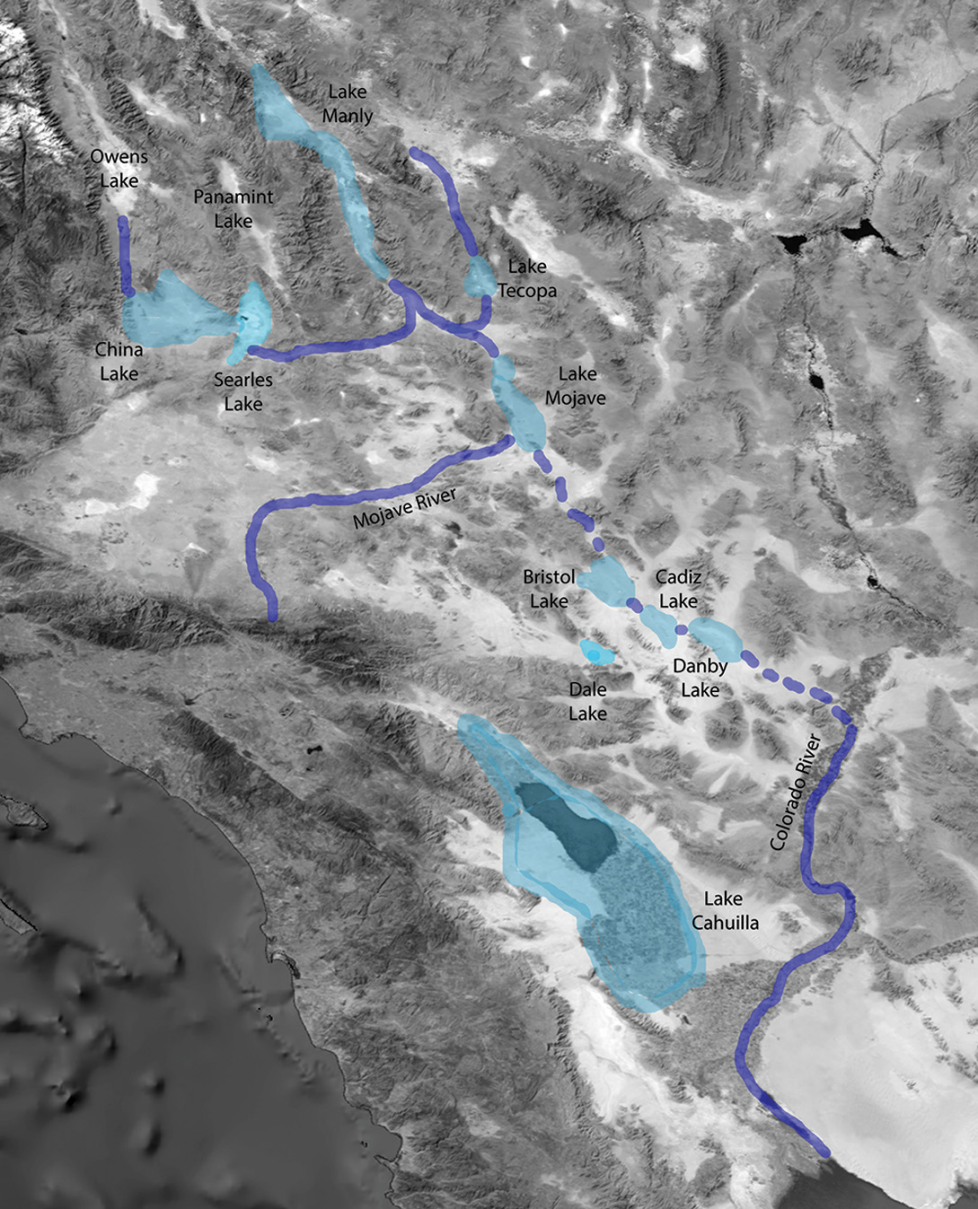
Southern California paleolakes (light blue) and paleorivers (dark blue) that are the focus of this study. Dashed dark blue lines indicate connections that are disputed by geological evidence. (Note: these are not to scale and not comprehensive regarding ancient lakes and rivers in this area; not all lakes would have been at their maximum sizes simultaneously, some lake basins dried up and re-filled multiple times, and there were more lakes present than those shown. The point of the figure is to provide a general idea of what the southwest was like before desertification).

In addition to overall changes in the environment, the climatic shifts have also altered corridors that previously connected populations across the landscape. For example, during the glaciopluvial periods of the Pleistocene, there were several large lakes in the Southwest that were periodically connected to each other by paleorivers or streams (Fig.1). Now, these dry lakes are typically covered with thick salt crusts, although during times of high rainfall, these lakes fill with water and may once again become connected to other dry lakes or drainage basins. Previous studies have established a correlation between the hydrographic history of the desert Southwest and the distribution and genetic structure of aquatic organisms such as springsnails and pupfishes (Hubbs and Miller 1948; Hershler 1989; Hershler and Pratt 1990; Echelle and Dowling 1992; Hershler et al. 1999), which are likely to have been strongly affected by episodes of aridification. However, whether or not this correlation extends to terrestrial species is unknown.

**Figure 2:**
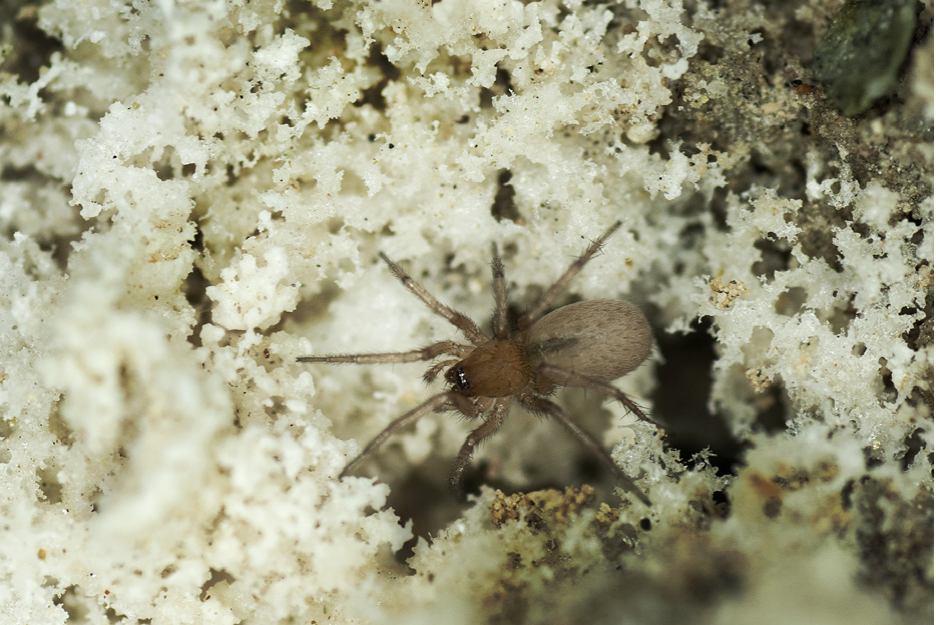
*Saltonia incerta* Banks, Soda Dry Lake, Zzyzx, California, cruising around on the salt crust, under which it lives in tiny sheet webs.

The current study examines population genetic variation across the geographic range of the small sheet-web-weaving spider *Saltonia incerta* Banks (Araneae: Dictynidae) (Fig.2). This species, the sole representative of the genus, is most closely related to species that are limited to aquatic habitats (Spagna et al. 2010). Not surprisingly, this desert-inhabiting species is limited to wet habitats. It is found on the underside of salt crusts on dry lakebeds or along the salty shores of wet or intermittent lakes and waterways in southwestern North America (Roth and Brown 1975; pers. obs.). Previously, the species was recorded from only three localities in the Mojave Desert (Roth and Brown 1975). The type locality (Salton, collected in 1897) remains submerged since the Salton Basin was flooded by the Colorado River in 1905. Similarly, a second habitat (Fish Springs, collected in 1941) has been transformed by human development. The exact locality of the third site (“Isla Pelicano”) is unknown (Roth and Brown 1975). Thus, the spider was, until recently, presumed to be extinct (Bennett 2005). However, in the 1990s, specimens were collected from fourth and fifth localities, China Lake and Soda Dry Lake in the Mojave Desert (D. Ubick & W. Savary, unpubl. data), adding to the four known specimens in museum collections. Additional recent (2002–2008) surveys in support of the current study located spiders in 10 new salt flat/intermittently wet localities that are all separated by several kilometers of desert.

**Figure 3:**
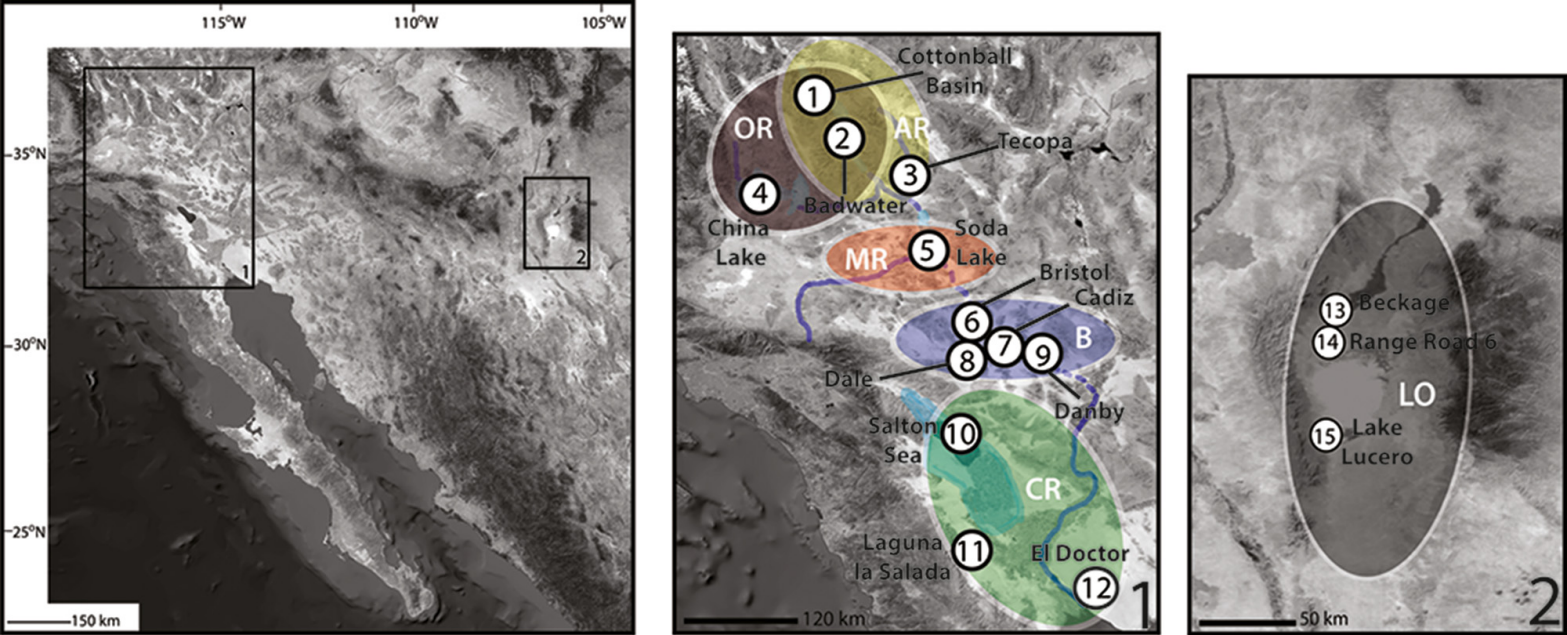
Map showing the southwestern United States. Areas enclosed in boxes on the left are the California sites (1) and the New Mexico sites (2) and are expanded on the right. White circles correspond to collection localities of *Saltonia incerta* used in this study. Numbers inside the circles correspond to locality numbers in Table1. The ellipses around the samples correspond to parts of drainage basins referred to in this study. Dashed dark blue lines indicate connections that may or may not have existed. OR, brown-Owens River, AR, yellow-Amargosa River, MR, red-Mojave River, B, blue-Bristol drainage, CR, green-Colorado River, LO, gray-Lake Otero.

The aim of this study is to determine whether there is gene flow between populations of this species and how this informs our understanding of the evolutionary history of the species in the context of the hydrographic history of the desert Southwest. We focus on the distribution and population structure of the spiders from the salt crust habitats of dry lakebeds and shores (hereafter collectively referred to as salt flats) contained within six drainages (including 10 new localities) in southwestern North America (Fig.3, Table1). Some of the salt flats and drainage basins were connected to each other in the past by paleorivers, although they are now isolated. Others are currently connected intermittently by rivers or streams, and some are not known to have ever been interconnected (Fig. 1).

**Table 1:** Specimen data, including specimen numbers, specific locality information and GenBank accession numbers. This also includes the salt flat, lake basin and drainage basin in which the collection locality is found. Specimen numbers correspond to salt_ voucher numbers. Locality numbers correspond to localities in Fig.3A ? after Mojave/Colorado indicates that it is unknown if any of the lakes in the Bristol drainage were actually part of either of these drainage basins, as discussed in the text

Specimen Numbers	Locality	Salt Flat	Lake Basin	Drainage Basin/Era of Existence	GenBank Accession Numbers
57, 59, 62	1) CA: Inyo Co., Death Valley National Park, Cottonball Basin off Hwy. 190; 36°30′49.64″ N 116°53′12.69″ W	Cottonball Basin	Lake Manly	Amargosa/Owens; Late Cenozoic and current	DQ411360, DQ411361, DQ411362
16, 89, 91	2) CA: Inyo Co., Death Valley National Park, Badwater, 36°13′15.09″ N 116°46′28.99″ W	Badwater	Lake Manly	Amargosa/Owens; Late Cenozoic and current	DQ411357, DQ411358, DQ411359
10, 23, 24, 63	3) CA: Inyo Co., off Hwy 127, ˜4 mi S of Shoshone, 35°54′58.85″ N 116°15′44.58″ W	Tecopa	Lake Tecopa	Amargosa; Late Cenozoic and current	DQ411341, DQ411342, DQ411343, DQ411344
80	4) CA: San Bernardino Co., China Lake, 35°41′58.61″ N 117°38′03.39″W	China Lake	China Lake	Owens; Late Cenozoic	DQ411335
1, 7, 9, 11, 12, 13, 21, 38, 45, 61, 94, 97, 102	5) CA: San Bernardino Co., Soda Dry Lake, Zzyzx35°09′34.97″ N 116°05′29.68″ W	Soda Lake	Lake Mojave	Mojave; Late Cenozoic	DQ411322, DQ411323, DQ411324, DQ411325, DQ411326, DQ411327, DQ411328, DQ411329, DQ411330, DQ411331, DQ411332, DQ411333, DQ411334
33, 34, 87	6) CA: San Bernardino Co., Bristol Lake off Amboy Rd., 34°27′30.04″ N 115°44′26.84″ W	Bristol Lake	Bristol drainage	Mojave/Colorado?; Late Cenozoic	DQ411383, DQ411384, DQ411385
19, 22, 37, 67, 79, 81, 90	7) CA: San Bernardino Co., Cadiz Lake, ˜8 mi. N of 62 near entrance to Tetra Lite Chemical, 34°15′19.36″ N 115°23′11.70″ W	Cadiz Lake	Bristol drainage	Mojave/Colorado?; Late Cenozoic	DQ411363, DQ411364, DQ411365, DQ411366, DQ411367, DQ411368, DQ411369
14, 15, 27, 35, 36, 42, 51, 53, 84, 100, 101, 104	8) CA: San Bernardino Co., Dale lake off Iron Age Rd., 34°08′08.68″ N 115°41′06.28″ W	Dale Lake	Bristol drainage	Mojave/Colorado?; Late Cenozoic	DQ411345, DQ411346, DQ411347, DQ411348, DQ411349, DQ411350, DQ411351, DQ411352, DQ411353, DQ411354, DQ411355, DQ411356
32, 60	9) CA: San Bernardino Co., Danby Lake, 34°13′45.00″ N 115°04′09.24″ W	Danby Lake	Bristol drainage	Mojave/Colorado?; Late Cenozoic	DQ411381, DQ411382
8, 17, 18, 26, 41	10) CA: Riverside Co., NE corner of the Salton Sea, near Salt Creek Beach, 33°26′48.98″ N 115°50′36.16″ W	Salton Sea	Salton Sea	Colorado; Late Cenozoic and current	DQ411336, DQ411337, DQ411338, DQ411339, DQ411340
29, 30, 52, 65, 66, 95, 103	11) Mex: BC: Laguna Salada 32°33′42.48″ N 115°43′38.45″ W	Laguna Salada	Laguna Salada	Colorado; Late Cenozoic and current	DQ411370, DQ411371, DQ411372, DQ411373, DQ411374, DQ411375, DQ411376
28, 31, 55, 98	12) Mex: Son: El Doctór off Sonora 003, 31°56′47.61″ N 114°43′56.56″ W	El Doctór	Shores along Colorado River, head of Gulf of California	Colorado; Late Cenozoic and current	DQ411377, DQ411378, DQ411379, DQ411380
82, 83, 86	13) NM: Sierra Co., WSMR, Beckage Site Playa, 33°16′11.38″ N 106°22′11.52″ W	Beckage Site	Lake Otero	Ancestral Rio Grande; Late Cenozoic	DQ411402, DQ411403, DQ411404
70, 71, 72, 73, 77	14) NM: Sierra Co., WSMR, Range Road 6, where Salt Creek crosses the road, 33°07′08.30″ N 106°23′13.55″ W	Range Road 6	Lake Otero	Ancestral Rio Grande; Late Cenozoic	DQ411397, DQ411398, DQ411399, DQ411400, DQ411401
68, 69, 74, 75, 76, 85, 88, 92, 93, 96, 99	15) NM: Doña Ana Co. WSNM, Lake Lucero, 32°41′51.92″ N 106°2′04.83″ W	Lake Lucero	Lake Lucero	Ancestral Rio Grande; Late Cenozoic	DQ411386, DQ411387, DQ411388, DQ411389, DQ411390, DQ411391, DQ411392, DQ411393, DQ411394, DQ411395, DQ411396

Three scenarios were examined regarding how populations may be structured following fragmentation caused by desertification across the entire region of study including (1) *Complete isolation* (2) *Dispersal during intermittent flooding*, and (3) *Aerial dispersal*. The *complete isolation* scenario is based on the premise that *Saltonia* is dispersal limited, but suitable habitats were more extensive during wetter times. As desertification occurred, the spiders retreated to areas where moisture persisted, such as underneath the salt crust. This scenario would lead to high genetic drift and divergence among populations in isolated drainage basins and between salt flats within drainage basins. At the local scale we do not expect structure within individual salt flats because of gene flow within contiguous habitat. The second scenario, *dispersal during intermittent flooding*, assumes that the spiders would disperse rarely (during flooding) and passively along waterways during wetter times. If this is true, population structure between drainage basins will be higher than within a basin because salt flats within a single drainage basin are more likely to become connected during an episode of flooding than salt flats from different drainage basins. Also, a pattern of isolation by distance might be expected because the spiders may not be able to travel far during the short periods of time the landscape is flooded. Finally, the *aerial dispersal* scenario postulates that gene flow occurs between both lake basins and drainage basins via aerial movements (some spiders balloon on silk threads, although it is unknown if *S. incerta* does this) without the need for corridors. The expectation in this scenario is low genetic structure (i.e., high levels of gene flow) both within and between basins, although prevailing winds and mountain barriers may lead to some directionality of movement.

## Methods

### Sampling sites

We examined twenty localities in six drainage (or paleodrainage) basins (1–4 sites sampled per drainage) that fit the macro- and micro-habitat constraints of the species. These habitats all contain moderate to thick salt crusts and are in the arid Southwest of North America. Of 20 localities examined, the spiders were found at 15 localities in six drainages as follows (detailed in Table1): (1) the *Amargosa River drainage*, where specimens were collected in two localities, one near the town of Shoshone on ancient Lake Tecopa and two in Death Valley. (2) The *Owens River drainage* – China Lake. (3) The *Mojave River drainage* – Soda Dry Lake. (4) The *Bristol drainage* – four geographically proximate dry lakes – Bristol, Cadiz, Danby and Dale – which are included in a single drainage, although it is unclear whether they have ever been connected to each other or to lakes in other basins. Some evidence indicates that Bristol Lake was connected to Cadiz Lake (Brown and Rosen 1995). (5) The *Colorado River drainage* where the spiders were found at three sites, the shores of the Salton Sea at Salt Creek, Laguna Salada and the El Doctór wetland. And (6) three sites on ancient Lake Otero in New Mexico – two in the White Sands Missile Range (WSMR) and one site from Lake Lucero in White Sands National Monument. Lake Otero is in the Tularosa Basin, into which the ancestral Rio Grande once drained, although the basin now only drains the surrounding mountains (Hawley 1993; Allen 2005).

### Specimen sampling

An attempt was made to collect a minimum of 10 spiders per locality, although this sample size was difficult to obtain at some sites. Collection localities are depicted in Fig.3 and detailed in Table S1. A unique number (e.g., salt_001-salt_119) was placed in each specimen vial (one spider per vial) that was used in this study. Specimens are deposited in the Essig Museum of Entomology at UC Berkeley, the American Museum of Natural History and the California Academy of Sciences.

Two species were sampled as outgroups, *Paratheuma armata* (Marples) (Desidae) and *Argyroneta aquatica* (Clerck) (Cybaeidae). Outgroup selection was based on the placement of *Saltonia incerta* in a higher level molecular phylogenetic study of related spiders (Spagna et al. 2010).

### Molecular analyses

DNA was extracted from a leg, or in the case of smaller specimens, several legs or the abdomen, using a Qiagen DNeasy (QIAGEN Inc., Venlo, Netherlands (corporate); Hilden, Germany (operational)) kit following the manufacturer's protocol. Because these populations were likely separated relatively recently, the fast-evolving cytochrome oxidase 1 gene (CO1) was chosen because previous studies (e.g., Crews and Hedin 2006) have shown that it evolves at a rate sufficient to represent recent divergence between populations of spider species. To assess congruence with the nuclear genome and divergence at a different time scale, we also used Histone 3 (H3) gene sequences.

Partial fragments of CO1 were amplified with the polymerase chain reaction (PCR) from 83 individuals of *S. incerta* and the two outgroup taxa using a touchdown protocol, Failsafe (EPICENTRE) PCR kit buffer ‘F’ and the primers ˜1718 salt (5′-TTTTTRTTATTTATTTCGTCTA-3′; this study) and 2776 spiderA (Vink et al. 2005) for a total of 840 base pairs. H3 was amplified with the primers H3aF and H3aR (Colgan et al. 1998) for a subsample of specimens and the two outgroup taxa from this larger dataset using a standard 3 step protocol of 35 cycles with an annealing temperature of 55°.

PCR products were purified using Qiagen QIAquick (QIAGEN Inc.) spin columns or Exosap-IT (USB Industries) following the manufacturers' protocols and sequenced in both directions using the same primers that were used for PCR on an ABI 3730 capillary sequencer. DNA sequences were edited using Sequencher v4.6b2 (Gene Codes Corporation, Ann Arbor, Michigan, USA) and aligned by eye in MacClade 4.0 (Maddison and Maddison 2001) using the amino acid translation. Sequences can be found on GenBank under the accession numbers provided in Table1.

### Phylogenetic and population genetic analyses

Bayesian phylogenetic analysis was performed using MrBayes v3b4 (Huelsenbeck and Ronquist 2001) to estimate tree topologies, model parameters and posterior probabilities of inferred clades. This method of phylogenetic analysis was chosen because of its computational tractability and ability to employ complex models of nucleotide substitution. Likelihood ratio tests (LRTs) were used as model choice criteria (MrModeltest: Nylander 2002). Three analyses were conducted, one using only the mitochondrial data, one using the nuclear data alone and one with the mitochondrial and nuclear data combined. Each gene and codon position was modeled separately because different codon positions within a gene undergo different rates of substitution, and previous studies indicate that partitioned analyses provide better estimates of phylogenetic reconstruction (Nylander et al. 2004; Brandley et al. 2005). We used the HKY, F81 and GTR + Γ models for the evolution of codon positions 1, 2 and 3 of CO1, and F81, JC and HKY models for codon positions 1, 2 and 3 of H3. Three replicates were conducted using random starting trees, default priors and four heated chains at the default temperature. To ensure convergence of likelihood values across replicates, average likelihood scores (-lnL) were examined. Each Bayesian replicate analysis was run for 7,000,000 generations, sampling every 100th tree. Using the “cump” command in the program AWTY (Are We There Yet?; Wilgenbusch et al. 2004), we found that posterior probabilities converged at approximately 3,000,000 generations in each analysis; the preceding generations were eliminated as burn-in.

We assessed levels of genetic differentiation in the CO1 gene by calculating sequence divergence among populations, *F*-statistics (*F*_ST_) and hierarchical analyses of molecular variance (AMOVA; Excoffier et al. 1992). All three methods were implemented in the program ARLEQUIN v.3.5 (Excoffier et al. 2005). Percent sequence divergence was calculated using pairwise differences. Pairwise *F*_ST_ compares levels of variation among populations, corrected for variation within populations, with significance assessed from 10,100 permutations. AMOVA analyses allow for examination of hierarchical patterns of variation among groups of populations (*F*_CT_), among populations within groups (*F*_SC_) and among individuals within populations (*F*_ST_). Several distance methods were used (pairwise, Tamura-Nei, K2P), and the results were compared. Significance was tested using permutations of the data set described by Excoffier et al. (1992). Populations were grouped according to both geographic distance and drainage criteria instead of solely by drainage because sampling in some drainages was low (e.g.-only one specimen from the Owens River paleodrainage (China Lake), or there was only one locality available within a drainage (e.g.-Soda Dry Lake in the Mojave drainage) (Table1). Thus, the data were partitioned into four groups: Owens, Mojave and Amargosa drainages; Bristol drainage; Colorado River drainage; and Lake Otero in New Mexico (Fig.3). These analyses allowed examination of the alternative scenarios of population history. Under a complete isolation scenario, we would expect comparisons among drainage basins and isolated salt flat populations to be significant; under a scenario of dispersal during intermittent flooding, we would only expect comparisons among drainage basins to be significant; and under a scenario of aerial dispersal, we would not expect any of the population comparisons to be significant. If terrestrial dispersal occurred after fragmentation, a pattern of isolation by distance would be expected, so we also conducted an isolation by distance analysis using IBDWS (Jensen et al. 2005) with 30,000 randomizations.

The results of the AMOVA and phylogenetic analysis prompted two further analyses. One was a second AMOVA partitioned as above but excluding specimens from New Mexico. The second analysis was conducted using the “isolation with migration” software package IMa2.0 (Hey and Nielsen 2004, 2007) to determine if migration was responsible for the lack of monophyly in the geographically proximate Bristol and Colorado drainage basins. This program uses Markov Chain Monte Carlo (MCMC) methods to fit to the data a model of isolation with migration between two closely related populations. Multiple iterations of the program were run using 20 chains with 29,999,971 steps following a burn period of 1,000,000. Convergence of the MCMC search algorithm was assessed by examining the Effective Sample Size (ESS) values, the parameter update rates and the similarity among runs. The migration parameters and their associated HPD95Lo and HPD95Hi values were used to calculate migration rate per year, and the divergence time was estimated from the divergence time parameter t using a molecular clock calibrated at 2.25% per million years. This substitution rate has been calibrated based on mitochondrial divergence patterns in other spiders (Bidegaray-Batista and Arnedo 2011).

## Results

### Specimen sampling

Specimens were collected from 15 sites, including 10 localities from which the species were previously unknown in California, northern Mexico and New Mexico (Fig.3, Table1). Spiders were not found in other areas of potentially suitable habitat, including: the Salt Spring Hills, Saline Valley, Searles, Koehn, Harper and Owens Lakes in California; and Mesquite Lake and Ash Meadows in Nevada.

### Molecular phylogenetic and population genetic analyses

**Figure 4:**
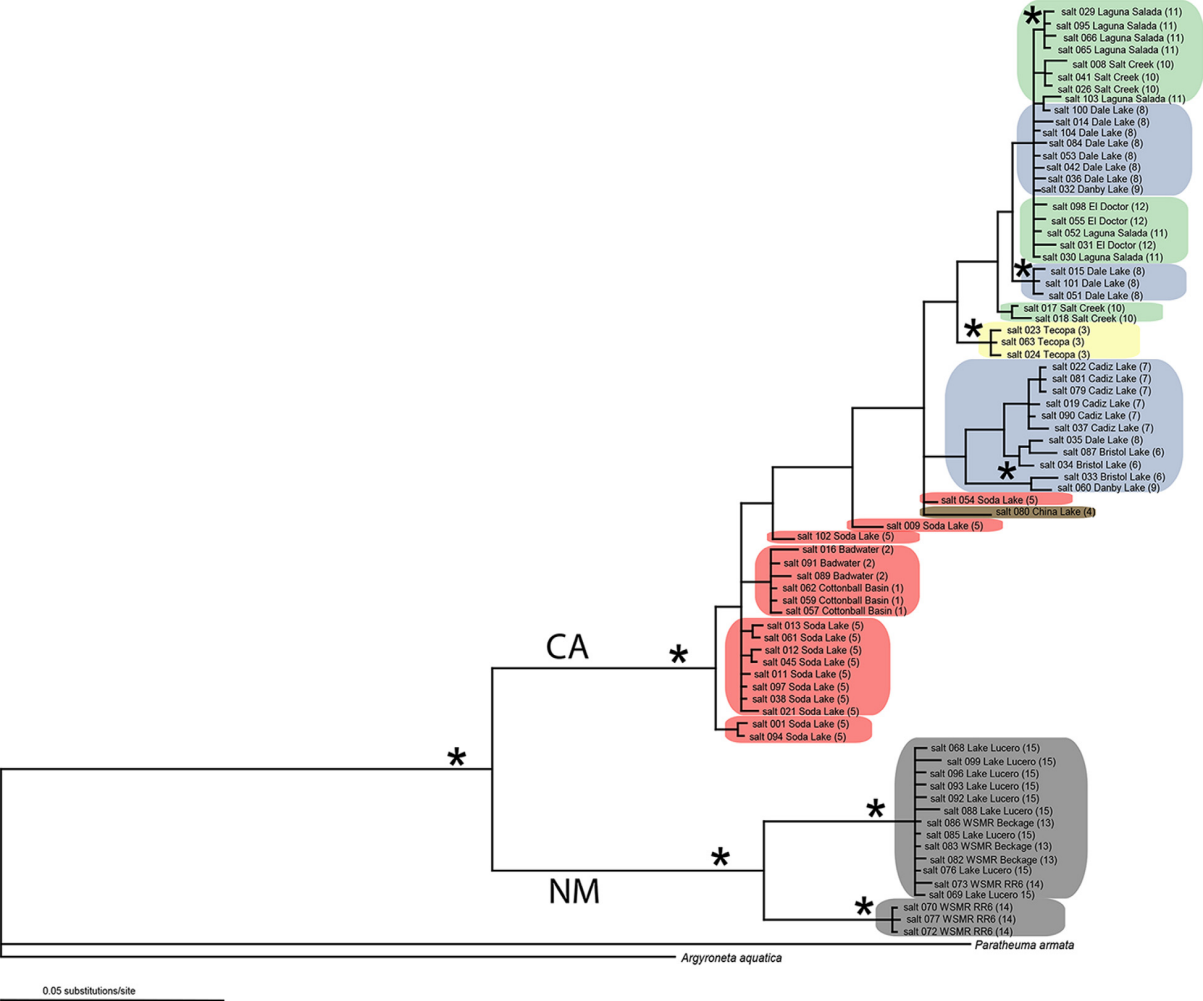
50% majority rule consensus phylogram from 120,000 trees. Asterisks denote nodes supported by >95% Bayesian posterior probabilities. Colors refer to drainage basin, and numbers following the site name correspond to those in Fig.3. LO, grey = Lake Otero, MR, red = Mojave River, OR, brown = Owens River, AR, yellow = Amargosa River, CR, green = Colorado River, B, blue = Bristol drainage.

A total of 41 unique CO1 haplotypes from 83 individuals plus two outgroup taxa were found. *Saltonia incerta* sequences form two well-supported (pp>0.95) major clades-a New Mexico (NM) clade and a California/Northern Mexico (CA) clade. These clades are also supported in the analysis of H3, although H3 provided no within-clade resolution. The 50% majority rule consensus phylogram from the combined analysis is shown in Fig.4. Although the New Mexico individuals were sampled from three geographically clustered areas on one ancient lakebed, they constitute two well-supported (pp>0.95) lineages. One consists of specimens from WSMR Range Road 6 (RR6), and a second consists of specimens from this same locality plus specimens from WSMR Beckage Site and Lake Lucero in WSNM (Fig.3, numbers 13–15). The percent pairwise sequence divergence of the CO1 locus is 1.8% for these two New Mexico lineages. Sequence divergence among populations in California (3.1%), and between New Mexico and California, is substantially higher (6.1%) (Table2). There is also a large divergence between the ingroup and outgroup. There is no conflict between the mitochondrial and nuclear DNA datasets, although the mtDNA locus is more variable.

**Table 2:** Percent pairwise sequence divergences

	Between the Ingroup and the Outgroup	Within New Mexico	Within California	Between New Mexico and California
CO1	14.8	1.8	3.1	10.5
H3	16.8	0.28	0.15	0.85
Combined	14.2	1.7	3.0	6.1

There are several subclades within the CA clade, indicating substantial population structure, although only four of them have high Bayesian posterior probability support. Only three localities (Death Valley, Tecopa, Cadiz Lake) are monophyletic, and only one of these (Tecopa) is well supported, whereas a single mitochondrial haplotype is found in four geographically widespread salt flats (Salt Creek near the Salton Sea, El Doctór, Dale Lake and Danby Lake) from two drainages (the Colorado River drainage and the Bristol drainage). Haplotype networks were constructed using TCS (Clement et al. 2000) and are shown in Figure S1.

Pairwise population divergence (*F*_ST_) ranged from zero at nearby sites to near fixation among basins within California (Tables S1 and S2). Large statistically significant differences in *F*_ST_ are evident between California and New Mexico sites. Although *F*_ST_ is large for all comparisons with China Lake, the values are not significant because of only a single sample from this site. The AMOVA indicates significant genetic variation at all levels, including among basins, salt flats within basins and individuals within salt flats (Table3). An AMOVA was also conducted that excluded the New Mexico localities to ensure that there is significant population structure within the CA populations (Table3). The results of this analysis remained significant, indicating that the molecular variance was not biased by the large genetic distance between the CA and NM populations. The results of the isolation by distance analysis indicated that there is no evidence of isolation by distance (Table S3).

**Table 3:** Results from the AMOVA. The data were partitioned into four groups: Owens, Mojave and Amargosa drainages; Bristol drainage; Colorado River drainage; and Lake Otero in New Mexico

	df	SS	Variance Components	% Of Variation	
Among groups (P) (incl. NM)	3	1378.88	19.77	67.66	F_CT_ 0.68 (<0.05)
Among populations within groups (P) (incl. NM)	11	373.13	6.54	22.39	F_SC_ 0.69 (<0.05)
Within populations (P) (incl. NM)	68	197.86	2.91	9.96	F_ST_ 0.90 (<0.05)
Among groups (P) (excl. NM)	2	419.60	7.13	40.67	F_CT_ 0.41 (<0.05)
Among populations (P) (excl. NM)	9	342.12	7.69	43.88	F_SC_ 0.74 (<0.05)
Within populations (P) (excl. NM)	52	140.89	2.71	15.45	F_ST_ 0.85 (<0.05)

**Figure 5:**
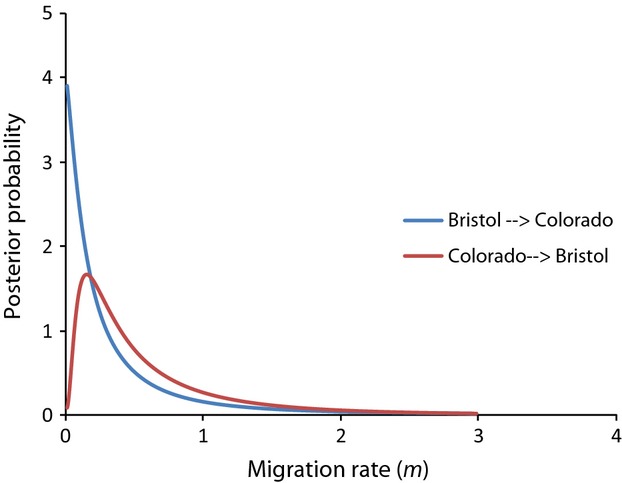
Plot of posterior probabilities from the IMa2 analysis show limited support for migration from the Bristol drainage to the Colorado drainage (blue line) and from the Colorado drainage to the Bristol drainage (red line).

Because of the similarity in the results from all IMa2 analyses and the ESS values being much greater than 50, the results from a single run are shown. IMa2 did not conclusively reject a model of no migration from Bristol to Colorado, but did reject a model of no migration from Colorado to Bristol. The results (Table4) indicate that the migration rates in either direction are very low, but are higher for migration from Colorado to Bristol (Fig.5). The divergence time estimates indicate that these populations have been separated from 893 to 1586 ky (Table4). The migration rate per year was also calculated, showing very low migration rates in both directions (Table4).

**Table 4:** Results from the IMa2 analysis

	*t*	*t* (years)	m0 > 1 (B→C)	m1 > 0 (C→B)	MRPY (B→C)	MRPY (C→B)
HiPt	16.88	893,122	0.0015	0.1455	2.835E-08	2.74995E-06
HPD95Lo	5.055?	257,460	0#	0#	0	0
HPD95Hi	29.98?	1,586,243	1.538#	1.825#	2.90682E-05	3.44925E-05

Results should be interpreted with caution given that HPD intervals may not be reliable (?) for parameter t, and that the results could change if the prior were changed for migration rates (#). However, when time is converted to years, the estimates are reasonable given the geological data, and we believe our prior for migration rate to be reasonable. In any case, migration rates are very low in either direction.

## Discussion

Genetic data were collected from across the range of the spider *Saltonia incerta* to provide evidence about patterns of gene flow among populations that inhabit salt flats in the desert southwest. In particular, three post-fragmentation scenarios were examined, including (1) complete isolation, (2) dispersal during intermittent flooding and (3) aerial dispersal via ballooning. The data revealed significant genetic structure across multiple spatial scales and suggest that population structure may have been shaped by fragmentation with isolation following the drying of the southwest.

The results of the AMOVA reveal genetic structure both within and between drainage basins. The gene tree reveals shared polymorphism between some drainage basins, and the results of the IM analysis indicate that migration is uncommon, so the shared polymorphism could be because of incomplete sorting rather than gene flow. The high levels of genetic structure suggest that ballooning does not play a major role in these spiders' dispersal, and this is consistent with isolation caused by large areas of unsuitable habitat separating desert salt flats: If the spiders were dispersing aerially, low genetic structure and directionality of movement because of prevailing wind patterns would be expected; however, genetic structure is high, and the minimal value of estimated gene flow from Bristol drainage to the Colorado drainage is not sufficiently high to be consistent with directional aerial dispersal. The data also favor complete isolation over dispersal during intermittent flooding because we found high genetic divergence among populations from isolated drainage basins and salt flats, but generally low population genetic structure within individual salt flats. If the spiders dispersed along waterways during rare times of flooding, we would expect that genetic structure within drainage basins would be less than between, which, according to the data shown here, is not always the case. Pairwise *F*_ST_ values can be large within basins, for example ranging from 0.16 to 0.75 within the Bristol drainage.

### Geological evidence and dispersal via intermittent waterways

Although most lineages within the California populations are not strongly supported by Bayesian posterior probabilities, the pattern of genetic divergence compliments natural geographical groupings. The Death Valley samples form a clade nested within the Soda Dry lake samples. This result is consistent with a historic connection that has been hypothesized to have linked Death Valley's ancient Lake Manly and Soda Dry Lake 186–120 kya (Hooke 2005; but see Knott 2005). Similarly, geological data show that there has never been a connection between Bristol Lake and the Mojave River (Brown and Rosen 1995; Rosen 2000), and the AMOVA analyses support this result. Spiders in the Bristol drainage are more genetically similar to each other than to spiders in any other basin, and there are no shared haplotypes between the Mojave River drainage populations and those from the Bristol drainage. If gene flow occurred recently from ballooning or overland travel, reduced genetic distance would be expected.

In Laguna Salada in northern Baja California and El Doctór in Sonora (both within the Colorado drainage basin), in which admixture of haplotypes is evident, the pattern could be attributed to historical gene flow. During the last few thousand years, the Colorado River has flowed into the Salton basin instead of directly into the gulf (Howard 1996). This would likely provide a larger area of wetland, allowing *Saltonia* to disperse more effectively across the region.

Some of the results indicate discrepancies between dispersal via intermittent waterways and geology. For instance, the phylogram (Fig.4) shows that many haplotypes are shared between Bristol, Danby and Dale Lakes, whereas Bristol and Cadiz Lake do not share haplotypes with one another. This pattern is not expected if gene flow occurs via aquatic corridors. Additionally, fossil evidence that Cadiz Lake may have briefly been connected with the Bouse Embayment (Smith 1970) is likewise not reflected in the *Saltonia* phylogram (Fig.4), because only Dale samples and a single Danby sample are grouped with samples related to the Bouse Embayment (El Doctór, Laguna Salada and Salt Creek), whereas the Cadiz samples are reciprocally monophyletic. Over a sufficient amount of time (measured in generations), it is expected that sequences from a single isolated locality would be more closely related to each other than to sequences from other localities if there is no gene flow. Therefore, the data are not congruent with a scenario in which Cadiz Lake was part of the Bouse Embayment with the exclusion of Dale or Danby because genetic admixture with individuals from Cadiz Lake and the other lakes would be expected instead of reciprocal monophyly.

### Aerial dispersal

There are three reasons to consider that ballooning is an unlikely or uncommon means of dispersal for *Saltonia*. (1) The IM results do not support high migration rates. (2) This species has never been observed to balloon, and it resides under the salt crust with few elevated habitat features to ascend to initiate the activity. Moreover, most of the salt flats are in valleys surrounded by mountains, which might make it more difficult for spiders to disperse aerially. Additionally, ballooning might be risky for spiders residing in such a specialized habitat, because the chance of finding salt flats subsequent to ballooning is unlikely. (3) The direction of the prevailing winds at any one site is largely inconsistent with the molecular results.

One way to examine the feasibility of ballooning without direct observation is to examine wind patterns, and whereas wind regime data from particular localities are not always available, most of the salt flats are associated with dunes and sand sheets, which can be used as a proxy for determining local wind pattern. In New Mexico, the dunes around Lake Lucero appear to be driven by winds from the west to southwest (Smith 1982), which is congruent with a ballooning scenario. That is, if wind patterns have been relatively consistent in the past several thousand years, it is possible that spiders may have blown to New Mexico from California or Northern Mexico. However, if this were the case, genetic differentiation between the NM and CA populations would be lower than that demonstrated here.

Prevailing winds in the Mojave Desert are typically from the west and the south (Lancaster and Tchakerian 2003). Therefore, migration would be expected to occur from the Colorado to the Bristol drainage; however, the IM results indicate very low migration rates in this direction. Additionally, there are variations in wind direction and intensity that are controlled by local topography (Smith 1982) or changes in intensity over long time periods (Smith 1967). The lakes in the Bristol Drainage lie in two sand transport paths, the Bristol Trough and Clark's Pass. These topographic features show an overall trend of sand blowing from west to east (from the Mojave to the Colorado River; Zimbelman et al. 1995), and there is little evidence that this has differed much in the past several thousand years (Smith 1982). If the spiders were able to become airborne, they would likely be carried eastward because of local topography, although eventually prevailing winds would take over (towards the northeast). Again, this pattern is not consistent with the results of the IM analysis or the phylogram. Therefore, if spiders are ballooning, they do not appear to follow the general pattern that is expected from what is known about local or prevailing winds in the Mojave Desert.

### Complete isolation

The idea that the populations of *Saltonia incerta* are isolated with little to no gene flow is supported by the phylogenetic analysis and AMOVA and is largely consistent with geological evidence. Under such a scenario, we predicted a pattern of high population structure between drainage basins (and salt flats within these basins), but low structure within individual salt flats because gene flow within salt flats is expected. These patterns are evident in the data. The exception to this pattern is in the Tularosa Basin in New Mexico, where proximate samples are genetically quite divergent, more so than on any of the salt flats to the west. However, rather than oppose the isolation scenario, the data from the New Mexico localities actually support it. The ancestral Rio Grande spilled into the Tularosa Basin via Fillmore Pass from the Pliocene to the Pleistocene (Hawley 1993; Allen 2005). Thus, out of the localities that were sampled, this locality would have been the first *Saltonia* locality to have been fragmented. The connections between the localities further west were more recent. Therefore, the reciprocal monophyly of the samples from New Mexico and the lack of monophyly that may be due to incomplete sorting in the more western populations are congruent with geological data and low-to-no gene flow. The genetically divergent yet geographically proximate samples found on ancient Lake Otero are also consistent with an isolation scenario because *Saltonia* are expected to be poor dispersers, and thus even spiders found near one another are not exchanging genes. We predict that similar patterns would emerge in the western populations had sufficient time elapsed since isolation.

### Comparison to other biological studies

To draw broader conclusions from the patterns in this dataset, the results can be considered in the context of other taxa inhabiting the same region and limited to similar habitats; in particular, the aquatic taxa, springsnails (Hershler and Sada 1987; Hershler 1989, 1999; Hershler et al. 1999) and pupfish (Echelle and Dowling 1992; Echelle and Echelle 1993; Echelle et al. 2005). In both groups, dispersal via aquatic routes is not unexpected. Indeed, it may seem inappropriate to compare the terrestrial *Saltonia* spiders to aquatic organisms. However, because we have examined two scenarios (complete isolation and dispersal via intermittent waterways) that rest on the assumption that waterways are a limiting factor for the spiders, we might expect them to show patterns similar to these aquatic organisms.

Springsnails are dispersal limited, with moisture being the limiting factor. Therefore, little or no gene flow between localities is expected. Although some studies have shown that the snails may disperse via waterfowl (Liu et al. 2003), the more general finding is that each species is geographically restricted and locally endemic to lake basins or drainages (Hershler and Sada 1987; Hershler 1989, 1999; Hershler et al. 1999). However, populations of one genus of springsnail (*Tryonia*) from the Amargosa drainage are not monophyletic (i.e.-do not have a single geographic origin) (Hershler et al. 1999). Whereas the overall high genetic structure is similar to *Saltonia*, populations of *Saltonia* found at salt flats in the Amargosa drainage (Death Valley and Tecopa samples) show a greater degree of genetic isolation than springsnails.

*Cyprinodon* pupfishes also rely on aquatic connections for dispersal and exhibit some concordance with patterns in *Saltonia*. Neither group is monophyletic in the Owens River drainage (Echelle and Dowling 1992; Echelle and Echelle 1993), whereas both show close relationships between the Owens River drainage and the Colorado drainage. However, all sampled fish from the Amargosa drainage are monophyletic, which contrasts with *Saltonia* populations in which specimens from Tecopa are distinct but not closely related to the Death Valley specimens. In both *Cyprinodon* and *Saltonia*, basal mitochondrial lineages are unresolved and incomplete lineage sorting is invoked to explain non-monophyly in *Cyprinodon* (Echelle et al. 2005).

Another terrestrial arthropod that justifies further study and occurs in the same salt flat habitats as *Saltonia* is the anthicid beetle *Tanarthrus* (Chandler 1975, 1979, 1984). These beetles are powerful flyers, yet are characterized by morphological diversity such that each isolated salt flat is home to an endemic species (Chandler 1975). Interestingly, in springsnails, pupfish and *Tanarthrus* beetles from this region, morphological divergence has occurred multiple times, but in *Saltonia*, even very geographically distant populations show no fixed morphological differences (unpubl. data). This could be because the snails, fish and beetles have diverged over longer periods and have had more time to accumulate differences or possibly because rates of morphological evolution are higher in these groups.

The patterns documented here are somewhat unusual in that most phylogeographic studies of non-ballooning spiders have found reciprocally monophyletic populations in geographically isolated sites (Hudson and Adams 1996; Bond et al. 2001; Hedin and Wood 2002). However, Masta (2000) found that incomplete lineage sorting was a better explanation than migration for a mitochondrial (16S-ND1) dataset for the jumping spider *Habronattus pugillis* on the basis that, as in *Saltonia*, closely related haplotypes were geographically separated.

### Conservation

*Saltonia* was recently thought to be extinct, but upon rediscovery in China Lake and Soda Lake in the 1990s, it was considered somewhat endangered (Bennett 2005). This study has expanded the previous knowledge of the distributional range of *Saltonia incerta*, indicating that it is likely a threatened species, rather than endangered. Several previous collecting sites have been altered by flooding and development, and they apparently no longer support populations of this species. It is unclear what proportion of the genetic diversity has been lost as a result of these alterations.

High levels of divergence suggest that populations are isolated and should be managed at the level of drainage basin. Because of the small number of populations in each basin, the potential impacts from continued habitat loss could be detrimental to *S. incerta*. Several of the areas where the spiders live are protected from major habitat alteration (e.g. Salton Sea State Park, Soda Lake in the Mojave National Preserve, El Doctor wetland, localities in Death Valley, Lake Otero in White Sands National Monument, White Sands Missile Range, China Lake). Other localities are used for salt mining (e.g.-KOH, NaOH) via evaporation. Whether or not this can harm populations is unknown, although typically only small portions of these dry lake beds are used for this purpose (though the entire lake area is relatively small). Although mandates forbid the driving of cars and OHVs across the surface of the lake beds, violations are frequent and detrimental effects of traffic across sensitive habitat has been demonstrated in dune ecosystems (Van Dam and Van Dam 2008). Clearly, long term population assessments are needed to determine how habitat disturbance affects the arthropod populations in these unique habitats.

## Conclusions

This study shows that *Saltonia incerta* is much more broadly distributed in salt flat habitats in southwestern North America than previously thought. Based on genetic data, we reject the hypotheses that the population structure of the spider is dictated by dispersal via intermittent waterways or by aerial ballooning. Instead, the data support a model of fragmentation followed by isolation of a formerly more widespread species. The geological evidence also argues for a once broader distribution of the spider because there is little congruence in the patterns of diversification of *Saltonia* with known paleoriver and paleodrainage data. Our results contrast with those of others who have examined populations and species that are restricted to paleoriver drainages, although most other studies have examined aquatic taxa in which gene flow is necessarily dictated by aquatic connections. Wetter conditions in the Southwest during the Pleistocene most likely contributed to a larger former distribution, whereas the subsequent desertification left populations restricted to salt flat habitats. Accordingly, the data are consistent with salt flats serving as refuges for relict populations from previous climatic periods, a finding that has been proposed for mesic oases in desert habitats (e.g., Grismer et al. 1994) but not for salt flats, which represent among the most physiologically demanding of habitats.

## Supporting Information

Figure S1 Haplotype networks.

**Table S1** Pairwise*F*_ST_ per site. **Table S2** Pairwise F_ST_ per basin. **Table S3** Results of isolation by distance analysis using IBDWS with 30,000 randomizations.
