# Supplementary material for: Desert salt flats as oases for the spider Saltonia incerta Banks (Araneae: Dictynidae)
Source: Ecol Evol. 2014 Sep 20;4(19):3861–74. doi: 10.1002/ece3.1242 (PMC4301052; doi:10.1002/ece3.1242)
Supplement: Supplementary file 2 — Table S1 PairwiseFST per site. Table S2 Pairwise FST per basin. Table S3 Results of isolation by distance analysis using IBDWS with 30,000 randomizations. [file ece30004-3861-sd2.docx]

Supplementary Figure 1. Haplotype networks. Numbers correspond to individuals in Table 1. Colors correspond to drainage basins: grey = Lake Otero, red = Mojave River, brown = Owens River, yellow = Amargosa River, green = Colorado River, blue = Bristol drainage. Squares indicated ancestral haplotypes, and the size of the square or oval corresponds to the number of haplotypes. A) Haplotypes that cannot be connected to any others. B) Haplotype network for the monophyletic Tecopa specimens. C) Haplotype network of 2 specimens from the Bristol drainage. D) Haplotype network of specimens from the Bristol drainage. E) Haplotype network of specimens from New Mexico. F) Haplotype network of specimens from the Mojave drainage. G) Haplotype network of specimens from the Bristol and Colorado drainages and a single specimen form the Mojave drainage.

Supplementary Table 1. Pairwise F_ST_ per site. An * indicates significance at the 0.05 level. The number in parentheses after the location refers to the numbers in Figure 2.

| Drainage | Amargosa / Owens | | |  |  | Owens | Mojave | Mojave / Colorado? | Mojave / Colorado? | Mojave / Colorado? | Mojave / Colorado? | Colorado | Colorado | Colorado | Ancestral Rio Grande | Ancestral Rio Grande | Ancestral Rio Grande |
| --- | --- | --- | --- | --- | --- | --- | --- | --- | --- | --- | --- | --- | --- | --- | --- | --- | --- |
|  | Cottonball Basin (1) | Badwater (2) | Tecopa (3) | | | China Lake (4) | Soda Lake (5) | Bristol Lake (6) | Cadiz Lake (7) | Dale Lake (8) | Danby Lake (9) | Salton Sea (10) | Laguna Salada (11) | El Doctor (12) | Beckage Site (13) | Range Road 6 (14) | Lake Lucero (15) |
| Cottonball Basin (1) | 0 |  |  | | |  |  |  |  |  |  |  |  |  |  |  |  |
| Badwater (2) | 0 | 0 |  | | |  |  |  |  |  |  |  |  |  |  |  |  |
| Tecopa (3) | 0.92* | 0.80* | 0 | | |  |  |  |  |  |  |  |  |  |  |  |  |
| China Lake (4) | 0.98 | 0.68 | 0.83 | | | 0 |  |  |  |  |  |  |  |  |  |  |  |
| Soda Lake (5) | 0.33* | 0.35* | 0.83* | | | 0.82 | 0 |  |  |  |  |  |  |  |  |  |  |
| Bristol Lake (6) | 0.71 | 0.59 | 0.63* | | | 0.24 | 0.75* | 0 |  |  |  |  |  |  |  |  |  |
| Cadiz Lake (7) | 0.98* | 0.90* | 0.92* | | | 0.96 | 0.86* | 0.52* | 0 |  |  |  |  |  |  |  |  |
| Dale Lake (8) | 0.75* | 0.72* | 0.52* | | | 0.60 | 0.78* | 0.59* | 0.75* | 0 |  |  |  |  |  |  |  |
| Danby Lake (9) | 0.66 | 0.51 | 0.42 | | | -0.30 | 0.74* | 0.01 | 0.71* | 0.16 | 0 |  |  |  |  |  |  |
| Salton Sea (10) | 0.90* | 0.81* | 0.71* | | | 0.82 | 0.83* | 0.68* | 0.91* | 0.01 | 0.25 | 0 |  |  |  |  |  |
| Laguna Salada (11) | 0.71* | 0.68* | 0.51** | | | 0.50 | 0.78* | 0.56* | 0.76* | 0.06 | 0.14 | 0.05 | 0 |  |  |  |  |
| El Doctor (12) | 0.96* | 0.86* | 0.83* | | | 0.93 | 0.86* | 0.74* | 0.96* | 0.02 | 0.34 | 0.20 | 0.06 | 0 |  |  |  |
| Beckage Site (13) | 0.78 | 0.74 | 0.80* | | | 0.59 | 0.87* | 0.70 | 0.89* | 0.83* | 0.61 | 0.82* | 0.75* | 0.81 | 0 |  |  |
| Range Road 6 (14) | 0.79* | 0.76* | 0.81* | | | 0.71 | 0.85* | 0.74* | 0.87* | 0.82* | 0.68* | 0.82* | 0.78* | 0.82 | 0.31 | 0 |  |
| Lake Lucero (15) | 0.95* | 0.93* | 0.95* | | | 0.95 | 0.93* | 0.92* | 0.96* | 0.90* | 0.91* | 0.94* | 0.89* | 0.95 | 0.32 | 0.57* | 0 |

Supplementary Table 2. Pairwise F_ST_ per basin. An * indicates significance at the 0.05 level.

|  | Amargosa | Owens | Mojave | Bristol Complex | Otero | Colorado |
| --- | --- | --- | --- | --- | --- | --- |
| Amargosa | 0 |  |  |  |  |  |
| Owens | 0.32 | 0 |  |  |  |  |
| Mojave | 0.32* | 0.82 | 0 |  |  |  |
| Bristol Complex | 0.33* | 0.18 | 0.60* | 0 |  |  |
| Otero | 0.75* | 0.76 | 0.82* | 0.76* | 0 |  |
| Colorado | 0.55* | 0.67 | 0.80* | 0.26* | 0.82* | 0 |

Supplementary Table 3. Results of isolation by distance analysis using IBDWS with 30,000 randomizations. Analysis 1 is a correlation between genetic similarity (M) and geographic distance. Analysis 2 is a correlation between M and log(geographic distance). Analysis 3 is a correlation between log(M) and geographic distance. Analysis 4 is a correlation between log(M) and log(geographic distance).

| New Mexico |  |  |
| --- | --- | --- |
|  | H_0_ r>=0 | H_0_ r<=0 |
| 1 | p=0.5030 | p=0.6593 |
| 2 | p=0.5006 | p=0.6692 |
| 3 | p=0.5006 | p=0.6692 |
| 4 | p=0.5006 | p=0.6692 |
| California |  |  |
| 1 | p=0.5551 | p=0.4499 |
| 2 | p=0.4324 | p=0.5676 |
| 3 | p=0.6228 | p=0.3772 |
| 4 | p=0.5649 | p=0.4351 |
